# On-Line Monitoring the Growth of *E. coli* or HeLa Cells Using an Annular Microelectrode Piezoelectric Biosensor

**DOI:** 10.3390/ijerph13121254

**Published:** 2016-12-18

**Authors:** Feifei Tong, Yan Lian, Junliang Han

**Affiliations:** 1 School of Biological and Chemical Engineering, Nanyang Institute of Technology, Nanyang 473000, China; 2 School of Chemistry and Materials Engineering, Huaihua University, Huaihua 418000, China; arline7419@hotmail.com; 3 School of Mechanical and Automotive Engineering, Nanyang Institute of Technology, Nanyang 473000, China; hanhanhgd@163.com

**Keywords:** annular microelectrodes, piezoelectric frequency, biosensor

## Abstract

Biological information is obtained from the interaction between the series detection electrode and the organism or the physical field of biological cultures in the non-mass responsive piezoelectric biosensor. Therefore, electric parameter of the electrode will affect the biosensor signal. The electric field distribution of the microelectrode used in this study was simulated using the COMSOL Multiphysics analytical tool. This process showed that the electric field spatial distribution is affected by the width of the electrode finger or the space between the electrodes. In addition, the characteristic response of the piezoelectric sensor constructed serially with an annular microelectrode was tested and applied for the continuous detection of *Escherichia coli* culture or HeLa cell culture. Results indicated that the piezoelectric biosensor with an annular microelectrode meets the requirements for the real-time detection of *E. coli* or HeLa cells in culture. Moreover, this kind of piezoelectric biosensor is more sensitive than the sensor with an interdigital microelectrode. Thus, the piezoelectric biosensor acts as an effective analysis tool for acquiring online cell or microbial culture information.

## 1. Introduction

In recent years, piezoelectric biosensors [1,2] have been applied in numerous fields, including microbiological culture assays or cell biology research. The signal module of the sensor is composed of two main parts, namely, the biological information recognition component (receptor) and the signal-converting component (transducer). The receptor is used to identify the physical or chemical information of cultured biological objects. With the development of microelectronic manufacturing technology and microsensors, many array microelectrodes have been used as the piezoelectric biosensor receptor. Among these, the interdigital microelectrode (IDME) exhibits more advantages than conventional electrodes, such as a micro-scale structure, a high steady-state current density, a low ohm/voltage drop, and a rapid response time and mass transfer between the electrodes [3,4,5]. Thus, an IDME can be used in the electrolyte solution system with a high impedance background to detect cell or microbial culture [6]. 

However, the IDME structure is composed of two parallel groups of tiny finger electrodes inserted with each other. Two main electrodes, which connect the fingers of the IDME, exist at the outer edge. Therefore, the electric field spatial distribution on the surface of the IDME is asymmetric upon operation [7,8,9,10]. This asymmetry causes the heterogeneity of the mass transfer and results in the increasing electrode impedance of the electrolyte solution under a high frequency oscillation circuit. The symmetry of the electric field spatial distribution should be improved to enable the mass transfer between only the adjacent and polar opposite microelectrodes during the detection process [11,12]. In this study, the electric field distribution of a microelectrode was simulated using the COMSOL Multiphysics analytical tool. In addition, the piezoelectric response characteristic of the sensor, which was connected serially with a microelectrode, was measured to investigate its effect on the sensor’s signal. Result showed that the response characteristic of the piezoelectric sensor with an annular microelectrode was more sensitive than that with the IDME. Furthermore, a novel piezoelectric biosensor connected serially with an annular microelectrode was constructed successfully for the continuous detection of *E. coli* culture or HeLa cell culture. The new piezoelectric biosensor acted as an effective analysis tool for acquiring online cell or microbial culture information.

## 2 Materials and Methods

### 2.1. Apparatus

**Figure 1:**
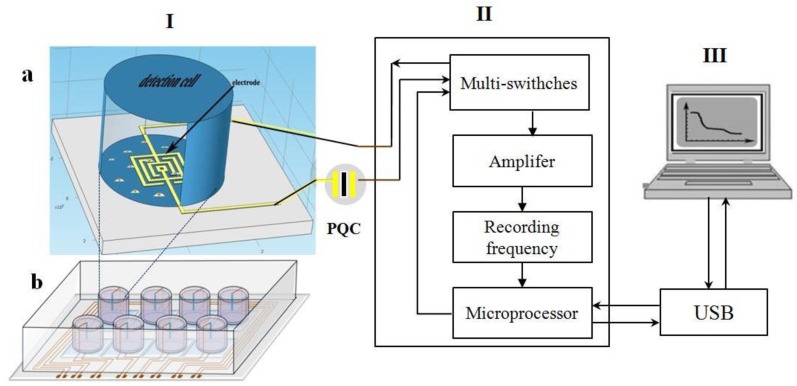
Schematic diagram of the piezoelectric biosensor connected serially with microelectrode. **I**: (**a**) culture-detection well; (**b**) a detection plate containing 8 detection wells; **II**: frequency meter and data processing system; **III**: PC interface; PQC: 9 MHz AT-cut piezoelectric quartz crystal.

The experimental instruments used in this work include an AT-cut 9 MHz piezoelectric quartz crystal (QCM, Beijing Jingyuxing Technology Co., Ltd., Beijing, China), a piezoelectric biosensor connected serially with a microelectrode (self-developed), whose structure is shown in Figure 1, an HP-4192A LF Impedance Analyzer (Hewlett-Packard, Palo Alto, CA, USA), an HHV Auto 500 magnetron sputtering apparatus (HHV, Bangalore, UK), a YM800 grinding polisher (Nanjiang Lisheng Co., Ltd., Nanjing, China), a KQ-500DV ultrasonic clearer (Kunshan Ultrasonic Instruments Co., Ltd., Kunshan, China), an SC-1B spin coater (Beijing Jinshengweina Technology Co., Ltd., Beijing, China), a hot plate (Beijing Jinshengweina Technology Co., Ltd., Beijing, China), an MA6 Mask aligner (SUSS MicroTec Group, Coventry, UK), a biological safety cabinet (Heal Force, Hongkong, China), a carbon dioxide incubator (Hunan Xianyi Instruments Co., Ltd., Changsha, China), and a CKX41 biological phase contrast microscope (Olympus, Takachiho, Japan). 

### 2.2. Materials and Reagents

Yeast extract culture detection of microorganism culture medium used with an series piezoelectric quartz crystal (SPQC) instrument was proposed in our laboratory. BP 212-37 positive UV Photoresist (Kempur Microelectronics Inc., Beijing, China), potassium chloride (KCl, Sinopharm Chemical Reagent Co., Ltd., Shanghai, China). *Escherichia coli* O157:H7, and HeLa cells were taken from the College of Biology, Hunan University, China. LB medium, DMEM medium, fetal bovine serum (FBS), trypsin, penicillin-streptomycin, and EDTA were purchased from Gibco, USA. The PBS buffer solution was composed of 8.1 mM Na_2_HPO_4_ + 136.7 mM NaCl + 2.7 mM KCl + 1.5 mM KH_2_PO_4_ (pH = 7.4). All other chemicals were of reagent grade. Ultrapure water (RN 18.2 MΩ/cm) was used throughout the experiment.

### 2.3. Design and Manufacture of the Annular Microelectrode

**Figure 2:**
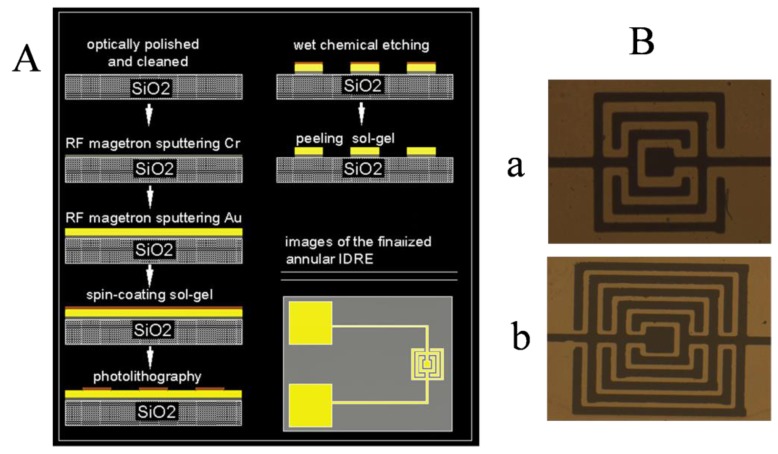
(**A**) Schematic diagram for the manufacture of the annular microelectrode; (**B**) Optical micrograph of the annular microelectrode. Black represents the Au microelectrode and yellow represents gaps or spaces. (**a**) Microelectrode with a 100 µm finger width and a 100 µm finger gap; (**b**) Microelectrode with a 100 µm finger width and a 50 µm finger gap.

The process of the annular microelectrode is shown in Figure 2A. SiO_2_ glass (with a thickness of 1.5 mm, and an area of 8 mm × 5 mm) was used as substrate of the annular microelectrode. We optically polished the glass surface and cleaned it with a piranha solution (1:3 (v/v) 30% H_2_O_2_/H_2_SO_4_) for 5 min. Then, the glass surface was sonicated in acetone and double-distilled water (each for 3–5 min) individually and dried under a nitrogen flow [13,14,15]. A 20-nm-thick Cr adhesion layer was first sputtered directly onto the glass surface via radio frequency magnetron sputtering, followed by a 200 nm gold layer as the microelectrode material on the Cr layer to enhance the adhesion of the Au layer on the glass substrate. Then, positive UV photoresist was evenly coated on the gold layer with a micro-annular shape [16]. After exposing the object to UV, we removed the unwanted photoresist and dissolved the corresponding Cr and Au films using wet chemical etching. The specially designed microelectrode with a lead line and bonding pad was obtained, and it was then sterilized with 75% ethanol and UV irradiated for 15 min for the experiment. The microelectrode shape is shown in Figure 2B.

### 2.4. Response Characteristics of the Piezoelectric Sensor with Microelectrode

The annular microelectrode was connected to the piezoelectric quartz crystal sensor in series. The frequency shift of the sensor was measured at different concentrations of a standard KCl solution. The detailed procedure is as follows: When the sensor’s microelectrode was immerged in purified water (i.e., the concentration of KCl is 0), the piezoelectric resonance frequency was recorded as F_(water)_ = F_0_. While replacing the water with KCl standard solution at different concentrations, the piezoelectric resonance frequency was recorded as F_(KCl)_ = F_i_ (*i* = 1, 2, 3…). Thus, the sensor’s response frequency shift could be obtained as follows: ΔF $=F_{i}-F_{0}$.

### 2.5. Detection of E. coli or HeLa Cells Using the Annular Microelectrode Series Piezoelectric Biosensor

For the detection of *E. coli* culture, 5 mL of LB medium was added to the culture-detection cell, and 1 mL of suspension containing 10^5^ cfu/mL of *E. coli* was mixed gently with the medium. Then, the annular microelectrode was inserted into the culture-detection cell. After connecting serially with the piezoelectric sensor detecting system, the *E. coli* suspension was incubated at 37 ± 0.2 °C. The response frequency shift was recorded automatically by the piezoelectric biosensor.

For the monitoring of HeLa cell growth, the annular microelectrode was assembled at the bottom of the culture-detection cell, which was used as the culture substrate. An amount of 1.5 mL of HeLa cell suspension at a concentration of 1 × 10^4^ cell/mL was seeded in the culture-detection cell and incubated in a carbon dioxide incubator under 5% CO_2_ at 37 °C and saturated humidity. The response frequency shifts, which indicated HeLa cell growth in real time, were recorded automatically by the sensor.

## 3. Result and Discussion

### 3.1. Multiphysics Simulation of the Microelectrode

The electric field spatial distributions of microelectrodes with different finger or gap widths in the medium were separately simulated under static conditions in three-dimensional (3D) space using COMSOL Multiphysics coupling analysis software [17]. The terminal voltage was set at 1 V. Voltage distribution was calculated using the following Poisson equation:

(1) $$-\nabla \cdot \left(\varepsilon_{r}\varepsilon_{0}\nabla V\right)=0$$

where ε0 is the dielectric constant in vacuum (8.86 × 10^−12^ F/m), and εr is the relative dielectric constant of the electrolyte solution.

The strength of the electric field spatial distribution in the simulation domain was described using the electric potential (E):

(2) E=−∇V

where ∇ is the Laplasse operator.

Equation (2) was used to calculate the charge conservation equation for the electric potential using the given spatial distribution of the electric charge. The equation is used primarily to model charge conservation in dielectrics under static conditions. The 3D simulation of the microelectrode was performed using these equations and the COMSOL program. 

**Figure 3:**
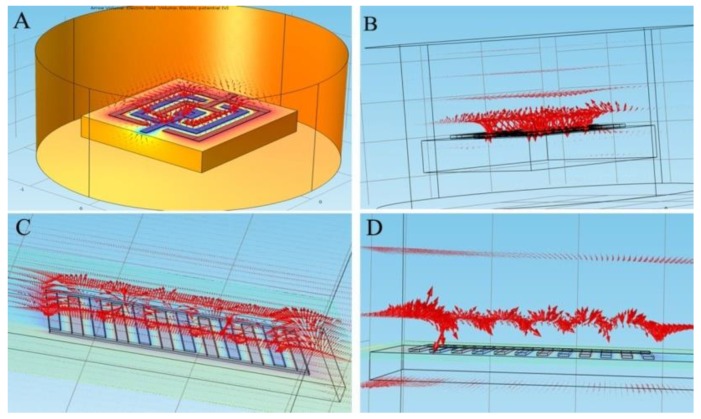
Electric field spatial distribution of the microelectrode under static conditions. (**A**) Electric field spatial distribution of the annular microelectrode; (**B**) Cross-sectional view of the annular microelectrodes; (**C**) Electric field spatial distribution of the IDME; (**D**) Cross-sectional view of the IDME.

Simulation results are showed in Figure 3. The arrow represents the electric line of force. In Figure 3, the large arrow indicates a greater density of the electric line of force and a stronger electric field. 

In the simulation of the microelectrode, on the upper spatial domain of the annular microelectrode, a closer distance between the microelectrode and the electrode surface, represents a greater change in the electric potential. Near the surface of the microelectrode, the electric line of force field intensity was very dense and displayed an annular shape, while the electric field weakened with increasing height of the electrode. Based on the simulation of the IDME, the electric field concentrated under the upper spatial domain of the interdigital electrode. However, the electric field intensity of the main electrode was stronger on the two sides and two terminals than those of the interdigital electrode, as influenced by the interdigital geometric structure. It is possible that, in the static field, the skin effect of the charge distribution resulted in the uneven distribution pattern of the electric field on the entire electrode surface. The mass transfer of electrolytes in the solution was then carried out in a large range. This phenomenon resulted in the poor transfer efficiency and electrochemical reaction sensitivity of the IDME sensor compared with the annular microelectrode sensor.

### 3.2. Influence of Annular Microelectrode Geometric Parameters on the Equivalent Circuit

The geometric parameters of the annular microelectrode are the band width, the gap size, and the number of annuluses of the electrode. We employed the simulation software to simulate the variation in the equivalent capacitance and equivalent resistance with those of the geometric parameters of the microelectrode.

**Figure 4:**
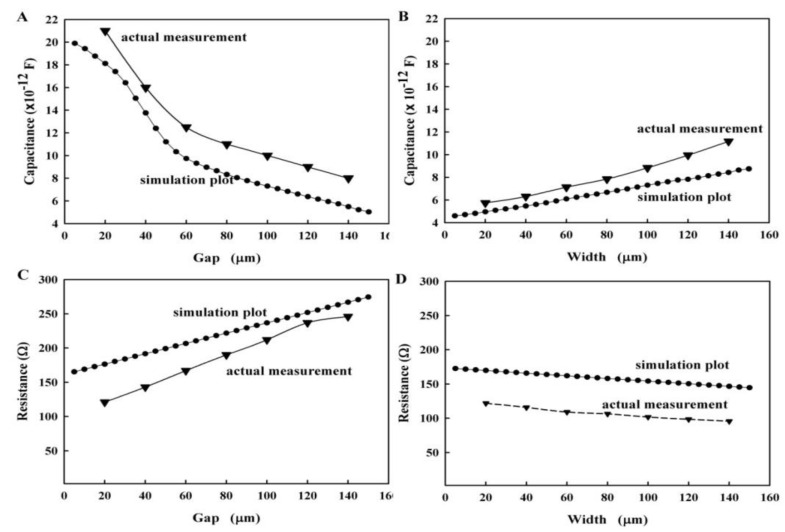
Influence of the geometric parameters of the electrode on the equivalent circuit. (**A**) The influence of the electrode gap on the equivalent capacitance; (**B**) Influence of the electrode band width on the equivalent capacitance; (**C**) Influence of the microelectrode gap on the equivalent resistance; (**D**) Influence of the microelectrode width on the equivalent resistance.

The annular microelectrode (shown in Figure 2A) was chosen as the electrode model. The influence of the electrode size on the equivalent capacitance and equivalent resistance was calculated using the simulation software. We set the electrode band width to 100 µm, and the electrode gap was changed from 5 µm to 150 µm with a step size of 5 µm. The electrode was applied with a 1 V AC voltage, and the frequency was 9 MHz. Results are shown in Figure 4. Results also suggested that the gap between the microelectrode bands had a direct impact on the equivalent capacitance. When the gap size increased from 5 µm to 55 µm, the equivalent capacitance decreased sharply. When the gap size was increased to 150 µm, the equivalent capacitance continued decreasing. However, the drop was reduced, as shown in Figure 4A. The equivalent resistance exhibited an upward trend as gap size increased, as shown in Figure 4C. Similarly, the influence of the electrode band width on the equivalent parameters is shown in Figure 4B,D. The increase in the annular microelectrode band width resulted in the increased linearity of the equivalent capacitance. However, the linearity of the equivalent resistance decreased, and the change in magnitude was smaller than the influence attributed to the changes in gap size caused by the increasing band width. Simulation results suggested that the influences of the microelectrode gap changes were greater than those of the microelectrode band width changes. 

In addition, the real capacitance and impedance of the annular microelectrode were measured using a 4192 impedance Analyzer at 9 MHz. As shown in Figure 4, the simulation results coincided with the measurement results. 

**Figure 5:**
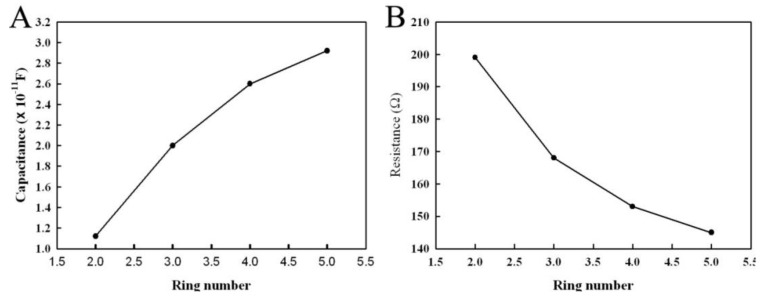
Influence of the number of annuluses on the electric parameters. (**A**) Influence of the number of annuluses on capacitance; (**B**) Influence of the number of annuluses on resistance.

In order to investigate the influence of the number of annuluses on the electric parameters, we designed the annular microelectrode with two, three, four, and five pairs of annuluses (every electrode pair with a 50 µm of electrode band width and 50 µm gap). The capacitance and resistance of the annular microelectrode were measured using the 4192A at 9 MHz. Results are shown in Figure 5. The changing curve of the electrode capacitance with the different number of annuluses is shown in Figure 5A. It suggests that the increase in annuluses of the annular microelectrode also increases the capacitance and resistance. This phenomenon might indicate that the increase in annuluses was due to the significant increase in the area of the microelectrode plate. Thus, the capacitance also significantly increased. On the contrary, as shown in Figure 5B, the electrode resistance significantly decreased with the increasing numbers of annuluses. Therefore, the increasing number of annuluses significantly increased the sensitivity of the biosensor, which was composed of the annular microelectrode and the piezoelectric quartz crystal. However, the substrate of the annular microelectrode was relatively small, and the number of annuluses was limited by the substrate area. Thus, the microelectrode with three pairs of annuluses was preferred for use in the piezoelectric biosensor. 

### 3.3. Frequency Response of the Annular Microelectrode Piezoelectric Biosensor

**Figure 6:**
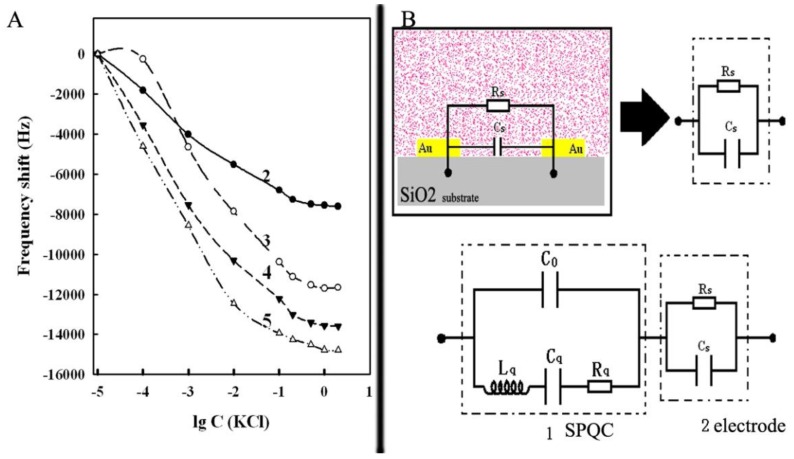
Response frequency of the annular microelectrode piezoelectric biosensor in KCl solution and the equivalent circuit model for the sensor. (**A**) Response frequency (curve 2: with 2 pairs annuluses; curve 3: with 3 pairs annuluses; curve 4: with 4 pairs annuluses; curve 5: with 5 pairs annuluses); (**B**) Equivalent circuit model of the sensor.

Annular microelectrode piezoelectric biosensors were constructed using two, three, four, and five pairs of annulus microelectrodes. Their piezoelectric frequency characteristics were studied at different concentrations of KCl solution. Results are shown in Figure 6. Findings indicated that both the absolute value of the piezoelectric frequency shift and the shift amplitude increased with increasing number of microelectrode annuluses. Thus, the increasing number of annuluses can improve the response sensitivity of the piezoelectric biosensor. However, the increased number of annuluses was not in accordance with the requirement or the microminiaturization and integration of the biosensor. Thus, three pairs or four pairs of annuluses were proposed for the construction of the annular microelectrode piezoelectric biosensor considering the two aspects mentioned.

The response frequency shift of the annular microelectrode piezoelectric biosensor was theoretically deduced as follows. For the convenient calculation, the equivalent circuit of the microelectrode and the piezoelectric crystal in series were simplified as shown in Figure 6B, where square 1 is the equivalent circuit of a piezoelectric resonance, and square 2 is the annular microelectrode. *C*_0_, *L*_q_, *C*_q_, and *R*_q_ represent the piezoelectric crystal static capacitance, the motional inductance, the motional capacitance, and the motional resistance, respectively. *C*_s_ is the equivalent capacitance of the annular microelectrode. *R*_s_ is the equivalent resistance of the solution. The equivalence circuit model showed the total complex impedance, Z, using the following equation:

(3) $$Z_{1+2}\;\;=\;\;j\frac{\left(\omega L_{q}-\;\frac{1}{\omega \;C_{q}}\right)}{1+\;\frac{C_{0}}{C_{q}}\;-\;\;\omega^{2}C_{0}L_{q}}\;\;+\;\;\;\frac{R_{s}}{1+\;\omega^{2}C_{s}^{2}R_{s}^{2}}\;-j\frac{\omega C_{s}R_{s}^{2}}{1+\;\omega^{2}C_{s}^{2}R_{s}^{2}}$$

where *ј* is the imaginary unit; *ω* is the angular frequency.

(4) $$Z_{1+2}=R\;\;+\;\;\;\;J\;X$$

where *R* is the real part of *Z*; *X* is the imaginary part of *Z*; *Ј* is the imaginary unit. According to the oscillation theory of the piezoelectric resonant phase shift condition, the phase shift of the feedback must be –θ and $–\theta =tan^{-1}\left(X/R\right)$.

*A* is defined by the equation $=\tan\theta =\frac{X}{R}$. Therefore, the following equation can be obtained:

(5) A+tan(−θ)=0.

By substituting the real and the imaginary parts of Equation (3) in Equation (5), Equation (5) can be transformed into Equation (6), as follows:

(6) $$\frac{AR_{S}+\omega C_{s}R_{s}^{2}}{1+{\left(\omega C_{s}R_{s}\right)}^{2}}-\frac{\omega L_{q}-\frac{1}{\omega C_{q}}}{1+\frac{C_{0}}{C_{q}}-\omega L_{q}C_{0}}=0.$$

Because ω=2πF and $F_{0}=\frac{1}{2\pi \sqrt{L_{q}C_{q}}}$, Equation (6) can be rewritten as Equation (7):

(7) $$F=F_{0}\left[1+\frac{{\pi F}_{0}C_{q}\left(2{\pi F}_{0}R_{s}^{2}C_{s}-{\mathrm{AR}}_{s}\right)}{1-2{\pi F}_{0}C_{0}R_{s}A+4\pi^{2}F_{0}^{2}R_{s}C_{s}\left(C_{0}+C_{s}\right)}\right].$$

For the annular microelectrode piezoelectric biosensor under the oscillating condition, Equation (7) must be differentiated as follows:

(8) $$dF=\frac{\partial F}{\partial R_{S}}dR_{s}+\frac{\partial F}{\partial C_{S}}dC_{s}.$$

$P_{1}$ and $P_{2}$ are defined by Equations (9) and (10):

(9) $$P_{1}=\frac{\partial F}{\partial R_{s}}=\pi F_{0}^{2}C_{q}\left\{\frac{A-4\pi^{2}F_{0}^{2}C_{s}^{2}R_{s}^{2}A-4\pi F_{0}C_{s}R_{s}}{{\left[1-2\pi F_{0}C_{0}R_{s}A+4\pi^{2}F_{0}^{2}R_{s}^{2}C_{s}\left(C_{0}+C_{s}\right)\right]}^{2}}\right\}$$

(10) $$P_{2}=\frac{\partial F}{\partial C_{s}}=2\pi^{2}F_{0}^{3}C_{q}\left\{\frac{1-4\pi^{2}F_{0}^{2}C_{s}^{2}R_{s}^{2}+4\pi F_{0}C_{s}R_{s}A}{{\left[1-2\pi F_{0}C_{0}R_{s}A+4\pi^{2}F_{0}^{2}R_{s}^{2}C_{s}\left(C_{0}+C_{s}\right)\right]}^{2}}\right\}$$

(11) $$dF=P_{1}dR_{s}+P_{2}dC_{s}$$

where *F*_0_ is the fundamental frequency of the piezoelectric crystal, *C*_0_ is the static capacitance of the crystal in Equations (9) and (10). They were constants. *R*_S_ and *C*_S_ are the initial resistance and the initial capacitance of the annular microelectrode, respectively. According to Equations (9)–(11), the frequency shift of the annular microelectrode piezoelectric biosensor was not only affected by the initial resistance and the initial capacitance of the annular microelectrode, but also by the changes in resistance and capacitance. The equivalent electric parameters of the sensor were detected using an HP-4192A Impedance Analyzer, with $P_{1}=2.31$ and $P_{2}=5.84\times 10^{12}$. Finally, Equation (11) can be transformed into Equation (12).

(12) $$\Delta F=2.31\times \Delta R_{s}+5.84\times 10^{12}\times \Delta C_{s}.$$

Therefore, as is evident from Figure 6A, when the concentration of the electrolyte solution is low, a slight increase in the KCl concentration can greatly increase the equivalent capacitance of the microelectrode. Thus, the piezoelectric frequency shift was significant. Moreover, the capacitance and resistance exhibited a downward trend when the concentration of KCl continuously increased. The piezoelectric frequency shift caused by the capacitance and resistance also decreased. Thus, when the sensor had increased the electrolyte concentration solution, the curve of the piezoelectric shift tended to be smooth. Significantly, the theoretical derivation coincides with the actual measurement results. 

### 3.4. Real-Time Monitoring of the Growth of E. coli HeLa Cells Using the Annular Microelectrode Piezoelectric Biosensor 

**Figure 7:**
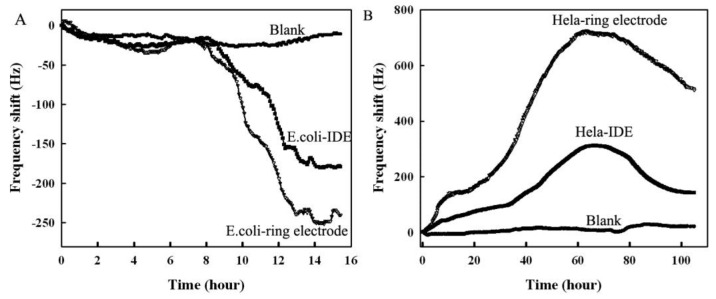
Real-time monitoring of the growth of *E. coli* or HeLa cells. (**A**) Real-time monitoring of the growth of *E. coli*; (**B**) Real-time monitoring of the growth of HeLa cells.

The microelectrode piezoelectric biosensor was employed to monitor the growth of *E. coli* and HeLa cell in real time. Results are shown in Figure 7. Both the annular microelectrode sensor and the IDME sensor can be used to monitor the growth of *E. coli* or HeLa cells. However, the maximal frequency shift signal of the annular microelectrode sensor was larger than that of the IDME sensor. This finding suggests that the piezoelectric biosensor series with the annular microelectrode possesses more response sensitivities. 

## 4. Conclusions

In this study, an annular microelectrode was designed and a novel piezoelectric biosensor in series was also constructed. Multi-physics field simulation software was employed to simulate and to calculate the electric field strength of the annular microelectrode. The simulated and calculated results suggested that the annular microelectrode has a very strong intensity electric field. Moreover, the electric field of the annular microelectrode exhibited an annular shape on the surface. The mass transfer of the electrolyte conducted within a large scope and the mass transfer efficiency between the microelectrodes were high because of the electric field shape distribution. Moreover, the influence of the geometric size of the annular microelectrode on the electrical parameters and the response frequency shift characteristics were investigated. The annular microelectrode piezoelectric biosensor, compared to the IDME piezoelectric biosensor, was more sensitive to the changes in the electrical parameters and more effectively monitors the growth of microorganism or mammalian cells in real time. Therefore, the piezoelectric biosensor series with an annular microelectrode is a promising detection method for detecting both microorganism with excellent sensitivity.
